# The Psychological Impact of In Vitro Fertilization (IVF): A Gender Systematic Review

**DOI:** 10.3390/healthcare14030375

**Published:** 2026-02-02

**Authors:** Maria Grammenou, Vasiliki Michou, Aikaterini Itziou, Arsenios Tsiotsias, Panagiotis Eskitzis

**Affiliations:** Department of Midwifery, School of Healthcare Sciences, University of Western Macedonia, Keptse, 50200 Ptolemaida, Greece; grammenou.maria@gmail.com (M.G.); aitziou@uowm.gr (A.I.); atsiotsias@uowm.gr (A.T.); peskitzis@uowm.gr (P.E.)

**Keywords:** in vitro fertilization, psychological impact, gender differences, infertility, mental health

## Abstract

Objective: In Vitro Fertilization (IVF) has revolutionized reproductive medicine, offering hope to individuals and couples facing infertility. However, the psychological impact of IVF varies significantly based on gender, necessitating a systematic review of the existing literature. This review explores the emotional effects of IVF on both men and women, highlighting gender-specific psychological responses throughout the treatment process. Methods: A systematic literature search using various databases (such as PubMed) was made. Studies published in English from the years 2000 to 2023 were included in the review. Results: A total of 47 studies examined the psychological impact of IVF on both women and men, covering the IVF programming period, the initial stages of IVF treatment, and subsequent stages, as well as the long-term psychological distress effects of IVF in both genders. Both female and male infertile patients are dealing with anxiety, depression and low quality of life. However, women were found to experience higher levels of psychological distress, including increased anxiety and depression symptoms, compared to men at nearly all stages of IVF treatment. Conclusions: Understanding these gender-specific differences is crucial for developing targeted psychological support interventions to improve mental well-being during IVF treatments.

## 1. Introduction

Infertility is a common yet often overlooked condition, affecting approximately 1 in 6 individuals globally, regardless of gender [1]. According to the World Health Organization (WHO) [2], evidence from studies conducted between 1990 and 2021 showed that infertility prevalence varies by region, with the Western Pacific Region reporting the highest lifetime infertility at 23.2%, followed by the Americas at 20.0% and Europe at 16.5%. Despite its high prevalence, awareness of fertility decline, associated risk factors, and available treatment options remains limited. Assisted reproductive technologies (ARTs), particularly in vitro fertilization (IVF), represent the most widely used medical approach to infertility and offer hope to many individuals and couples seeking parenthood [1,3].

Beyond its medical dimensions, infertility constitutes a major psychosocial stressor. The IVF process can heighten psychological strain due to its invasive procedures, physical discomfort, financial burdens, and uncertainty about treatment outcomes [4,5]. IVF treatment has been shown to increase emotional distress in women experiencing fertility issues during various stages of the IVF process, compared to those who do not face infertility in their journey to parenthood. Research indicates that the most stressful times within the IVF cycle are the oocyte retrieval and the waiting period before the pregnancy test [4]. Additionally, recent evidence indicates that women undergoing IVF experience a wide range of psychological burdens related to infertility, extending beyond average levels of distress. In a person-centered study, Liu et al. [6] identified distinct stress profiles among these women, categorized as low, moderate, and high stress. Those in the higher stress categories reported significantly greater psychological distress. Notably, partner attachment styles, specifically attachment anxiety and avoidance, partially or fully mediated the relationship between infertility-related stress and psychological distress, underscoring the importance of relational dynamics in understanding how IVF-related stress impacts mental health.

Women undergoing IVF consistently report higher levels of psychological distress than women who conceive without medical assistance, particularly in terms of anxiety, depressive symptoms, and reduced quality of life during treatment [7,8,9]. This elevated vulnerability is further supported by a large meta-analysis by Nik et al. [10], including 124,556 women, which demonstrated that women experiencing infertility have up to a 1.6-fold higher risk of psychological distress compared with women in the general population. It is also crucial to recognize that the psychological impacts of IVF are not limited to women. Men also grapple with short- and long-term emotional consequences, underscoring the need for a comprehensive understanding of the psychological dimensions of infertility and its treatment. Recent evidence by Li et al. [11] showed that both women and men undergoing IVF experience elevated levels of psychological distress, including anxiety and depressive symptoms, although women consistently report higher distress levels. Taken together, these findings indicate that infertility represents a shared psychosocial challenge within couples and highlight the importance of addressing the mental health needs of both partners throughout the IVF process.

Several reviews have previously addressed psychological distress associated with infertility and IVF; however, many focus predominantly on women [10,12], examine specific stages of treatment, or do not systematically compare psychological outcomes between genders across the full IVF trajectory [13]. For example, meta-analyses have highlighted higher distress in women compared with men but also noted variability in measurement and limited coverage of psychosocial mechanisms across treatment stages [14,15]. Moreover, prior psychosocial reviews emphasize individual risk factors and coping traits but do not integrate recent evidence across evolving ART practices and long-term outcomes [10].

The present systematic review aims to address these gaps by providing a comprehensive, gender-focused synthesis of the psychological impact of IVF across different stages of treatment, including pre-treatment planning, active treatment phases, and longer-term outcomes. By integrating evidence from both women and men and highlighting stage-specific patterns of anxiety, depression, and quality-of-life changes, this review seeks to clarify gender differences in psychological responses to IVF and to inform the development of targeted psychosocial support interventions for individuals and couples undergoing fertility treatment.

## 2. Materials and Methods

### 2.1. Protocol, Registration, and Institutional Approval

This systematic review was conducted according to a prespecified protocol that was submitted for registration in the PROSPERO database (submission number: CRD420261279713). At the time of manuscript submission and revision, the protocol was under review and had not yet been assigned a formal PROSPERO registration number. The study protocol was reviewed and approved by the University of Western Macedonia, Department of Midwifery, Ptolemaida, Greece (protocol number: 1512, approval date: 2 June 2022).

### 2.2. Literature Search Strategy

A systematic literature search was conducted across MEDLINE (via PubMed), PsycINFO, EMBASE, and Scopus to identify eligible studies for inclusion in this review. The electronic database searches covered the period from 1 January 2000 to 1 January 2024 and were last updated on 10 January 2024. The full search strategies for all databases are provided in the Supplementary Material (Tables S1–S4). To conduct the research, MeSH terms, keywords, and free words, such as “infertility AND in vitro fertilization OR sperm injections,” as well as “intracytoplasmic AND psychologist* OR anxiety OR depression OR emotions OR stress,” were employed in the search. Studies were included if they examined psychological outcomes, such as anxiety, depression, stress, or quality of life, in adult women and/or men undergoing IVF, had observational designs (cross-sectional, cohort, or longitudinal), were published in English between 2000 and 2023, and provided gender-specific or comparable data. To be more precise, gender differences were defined as differences in psychological outcomes between women and men undergoing assisted reproductive treatments. Studies were eligible if they reported psychological outcomes separately for women and men and provided data that allowed gender-related comparisons, even if sex-disaggregated results were not the primary focus of the study.

In contrast, studies were excluded if they met any of the following criteria: they were written in a language other than English, they were focusing exclusively on a single sex, adult women or men undergoing IVF, focused exclusively on other assisted reproductive modalities (such as intrauterine insemination or ovulation induction), did not address the psychological aspects of IVF treatment, had unclear measurement points or relationships between psychological factors and outcomes, employed randomized controlled trial designs, or were categorized as reviews, editorials, case reports, or conference abstracts. Randomized controlled trials were excluded from this review because they are primarily designed to evaluate the efficacy of psychological or behavioral interventions, rather than to describe the natural course and prevalence of psychological distress associated with IVF. The present systematic review aimed to synthesize observational evidence on gender-specific psychological responses across different stages of IVF treatment, focusing on naturally occurring experiences rather than experimentally manipulated interventions. Consequently, non-interventional study designs were considered more appropriate to address the objectives of this review.

In addition, a literature review of additional relevant articles was conducted to cover the theoretical background of this systematic review (infertility, assisted reproduction, etc.). Most eligible studies were collected from the PubMed and Scopus databases.

Initially, the PubMed database was utilized to perform systematic literature search, followed by the other databases. The search strategy, of which 66 articles emerged, used the following query stages and terms:

#1”fertilization in vitro”[MeSH Terms] OR “sperm injections, intracytoplasmic”[MeSH Terms]

#2 ((((((((((((((Fertilization* in Vitro[Title/Abstract]) OR In Vitro Fertilization*[Title/Abstract]) OR Test-Tube Fertilization*[Title/Abstract]) OR (Fertilizations [Title/Abstract] AND Test-Tube[Title/Abstract])) OR (Fertilization* [Title/Abstract] AND Test-Tube[Title/Abstract])) OR Test Tube Fertilization*[Title/Abstract]) OR Test-Tube Bab*[Title/Abstract]) OR (Bab* [Title/Abstract] AND Test-Tube[Title/Abstract])) OR Test Tube Babies[Title/Abstract]) OR (Sperm Injection* [Title/Abstract] AND Intracytoplasmic [Title/Abstract])) OR IVF[Title/Abstract]) OR (Injection* [Title/Abstract] AND Intracytoplasmic Sperm [Title/Abstract])) OR Intracytoplasmic Sperm Injection*[Title/Abstract]) OR (Injections [Title/Abstract] AND Sperm Intracytoplasmic [Title/Abstract])) OR ICSI[Title/Abstract]

#3 #1 OR #2

#4 ((((Psychology[MeSH Terms]) OR Anxiety[MeSH Terms]) OR Depression[MeSH Terms]) OR Emotions[MeSH Terms]) OR Stress, Psychological[MeSH Terms]

#5 ((((((((((((((((((Side Effect *[Title/Abstract] AND Psychological [Title/Abstract])) OR Psychological Side Effect*[Title/Abstract]) OR Anxiet*[Title/Abstract]) OR Depressi*[Title/Abstract]) OR Emotion*[Title/Abstract]) OR Distress*[Title/Abstract]) OR Psychological Stresses[Title/Abstract]) OR Stress*[Title/Abstract]) OR (Stress* [Title/Abstract] AND Psychologic*[Title/Abstract])) OR Psychologic* Stress*[Title/Abstract]) OR (Stress*[Title/Abstract] AND Life[Title/Abstract])) OR Mental Suffering[Title/Abstract]) OR (Suffering[Title/Abstract] AND Mental[Title/Abstract])) OR Suffering[Title/Abstract]) OR Emotional Stress[Title/Abstract]) OR (Stress [Title/Abstract] AND Emotional[Title/Abstract])) OR psychosocial[Title/Abstract]) OR psycholog*[Title/Abstract]

#6 #4 OR #5

#7 Infertility[MeSH Terms]

#8 ((((((Sterility [Title/Abstract] AND Reproductive [Title/Abstract])) OR Sterility[Title/Abstract]) OR Reproductive Sterility[Title/Abstract]) OR Sub-Fertility[Title/Abstract]) OR Subfertility[Title/Abstract]) OR Infertil*[Title/Abstract]

#9 #7 OR #8

#10 #3 AND #6

#11 ((((((((“randomized controlled trial”[Publication Type]) OR “controlled clinical trial”[Publication Type]) OR “ramdomized”[Title/Abstract]) OR “ramdomised”[Title/Abstract]) OR “placebo”[Title/Abstract]) OR “sham”[Title/Abstract]) OR “randomly”[Title/Abstract]) OR “trial”[Title/Abstract])

#12 #10 NOT #11

#13 (animals[MeSH Terms] NOT (humans[MeSH Terms] AND animals[MeSH Terms]))

#14 #12 NOT #13

#15 (“2000”[Date - Publication]: “3000”[Date - Publication])

#16 #14 AND #15

#17 English[Language]

#18 #16 AND #17

#19 (((man) AND (woman)) OR (men)) AND (women)

#20 #18 AND #19

### 2.3. Data Extraction

Two independent reviewers conducted the literature search and screened titles, abstracts, and full texts for eligibility. The reviewers were members of an independent statistical group from a different department of the university and were not involved in the conceptualization of the study. During the screening and data extraction process, they worked independently and assessed the articles using anonymized materials provided electronically, without direct access to author or journal identifiers beyond what was necessary for eligibility assessment. Communication with the research team was conducted exclusively via email and limited to first-name identification. The reviewers were not informed about the study hypotheses or planned publication outcomes at the time of screening. Discrepancies were resolved through discussion, and when consensus could not be reached, a third reviewer was consulted.

### 2.4. Risk of Bias Assessment

The risk of bias in all included non-randomized studies was assessed by two reviewers (M.G. and V.M.) using the Newcastle–Ottawa Scale (NOS). To be more precise, for cohort studies, risk of bias was assessed using the standard NOS, whereas for cross-sectional studies, a modified version of the NOS adapted for cross-sectional designs was applied [16,17]. The NOS is a star-based tool that evaluates methodological quality across three domains: selection of study groups, comparability of groups, and assessment of outcome or exposure, with a maximum possible score of nine stars. Stars indicate the number of criteria fulfilled within each NOS domain, with higher star counts reflecting better methodological quality. Discrepancies in scoring arose in three studies and were resolved through discussion. If consensus could not be reached, a third reviewer was consulted (P.E.). Studies that scored 7 to 9 stars were classified as having a low risk of bias; those scoring 4 to 6 stars, a moderate risk; and those scoring 0 to 3 stars, a high risk of bias [17]. The detailed risk-of-bias assessment for all included studies is presented in Results section.

## 3. Results

### 3.1. Literature Search

**Figure 1:**
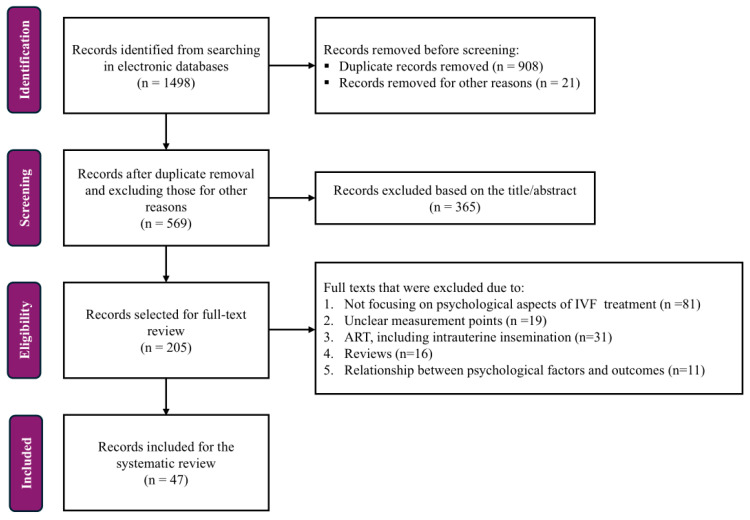
PRISMA flow diagram. IVF = In vitro Fertilization, ART = Assisted Reproductive Technology.

The comprehensive literature search yielded a total of 1498 records, of which 908 and 21 were removed due to duplication and for other reasons, respectively. Then, the remaining 569 records were screened for eligibility for the title and the abstract. After careful evaluation, 365 records were excluded as they did not meet the inclusion criteria. Lastly, 205 records were selected for full-text review, of which 158 were excluded due to not focusing on psychological aspects of IVF treatment (n = 81), unclear measurement points (n = 19), ART, including intrauterine insemination (n = 31), review type of article (n = 16) and the relationship between psychological factors and outcomes (n = 11). As a result, 47 research articles were included in the systematic review (Figure 1). This systematic review complies with the PRISMA guidelines.

### 3.2. General Characteristics of the Selected Studies

A total of 47 studies were included in the analysis. Most of these studies were conducted in Europe (n = 25), with Sweden leading the way (n = 8) and Italy in second place (n = 5). North America contributed 7 studies, followed by Israel with 3, South America with 2, and Iran with 2 as well. Additionally, there was 1 study each from Australia, Jamaica, Thailand, Turkey, China, and the United Kingdom. Notably, only one study was found to be international. Moreover, regarding the type of study, most of the studies were cross-sectional (n = 28) and longitudinal (n = 9) studies, compared to cohort studies (n = 4). These studies had a sample size for couples ranging from 19 to 1000, infertile women ranging from 19 to 1090, and infertile men from 17 to 1090. Twenty-one studies included both female and male infertility patients without referencing any relationship [18,19,20,21,22,23,24,25,26,27,28,29,30,31,32,33,34,35,36,37,38], while 25 studies enrolled infertile couples [9,39,40,41,42,43,44,45,46,47,48,49,50,51,52,53,54,55,56,57,58,59,60,61].

### 3.3. Programming an IVF Cycle or ART

Seven studies examined the psychological impact of infertility during the stage of planning or programming a future IVF cycle or other ART procedures [22,23,26,29,30,35,60], including two studies that focused explicitly on couples [22,60]. These findings indicate that the planning phase is marked by increased psychological vulnerability, with significant gender differences already apparent before the initiation of treatment. Detailed study characteristics and outcome measures are presented in Table 1.

#### 3.3.1. Psychological Distress During the Planning Stage

Across 2 studies, the planning stage of IVF or ART was associated with elevated levels of psychological distress, particularly anxiety, stress, and infertility-related concerns linked to uncertainty about treatment outcomes and decision-making demands. Women consistently reported higher levels of infertility-related anxiety and emotional distress than men, a pattern observed across different assessment tools and study designs (Table 1). Two studies further showed that women experienced greater social, sexual, and relationship-related anxiety. In contrast, men reported lower overall distress but remained vulnerable in specific domains such as social functioning and perceived role expectations [29,30]. Moreover, these studies—which also examined coping styles and stress management—highlighted that maladaptive strategies, including avoidance, self-blaming, and passive coping, were associated with higher depressive symptoms and stress levels in both genders. Alternatively, adaptive strategies such as problem-focused coping and social support seeking were linked to reduced psychological burden [29,30]. Notably, longitudinal comparisons indicated an increase in perceived stress over time among couples preparing for IVF, accompanied by a marked decline in the use of effective stress management strategies, suggesting a progressive accumulation of psychological strain even before treatment initiation [26].

#### 3.3.2. Quality of Life and Psychosocial Functioning

Several studies assessing quality of life during the IVF planning stage have consistently reported that women experience lower quality of life compared to men [22,58]. Women often face poorer emotional well-being and greater disruption in their daily functioning, which reflects the emotional demands and anticipatory stress associated with infertility and treatment planning. Conversely, men tend to have higher overall quality-of-life scores, although they still experience noticeable psychosocial strain. Additionally, various studies have identified broader psychosocial challenges during this phase, such as impaired couple adjustment, maladaptive coping strategies, elevated perceived stress, and irrational beliefs about parenthood. These factors can further compromise emotional well-being and interpersonal functioning [23,26,29,35].

#### 3.3.3. Gender Differences and Key Patterns

A consistent pattern across studies was the presence of marked gender differences in psychological responses during the IVF planning phase. Women tended to engage more intensively in infertility-related activities, such as information seeking, reflective thinking, and emotional disclosure, behaviors that were associated with higher stress levels and reduced quality of life [22]. On the other hand, men generally reported lower overall distress; however, they experienced increased stress when engaging in prolonged reflective thinking or when adaptive coping strategies were limited [22,30]. Further, evidence indicated that younger individuals planning IVF faced greater psychological and social challenges compared to the general population, highlighting their increased vulnerability [23].

### 3.4. Pre-IVF Psychological Reactions with or No Post-IVF Evaluation

Five studies examined psychological reactions immediately before the initiation of IVF treatment or at the very beginning of an IVF cycle [33,47,48,51,57]. Three of these studies also included post-treatment assessments, allowing examination of changes in psychological status over time and in relation to treatment outcomes [47,48,57]. Most studies included both women and men, either as individuals or as couples, and their main findings are summarized in Table 2.

#### 3.4.1. Psychological Distress Before IVF Initiation

Across 3 studies, the pre-IVF period was marked by significant psychological distress. Women consistently reported higher levels of anxiety, depression, and infertility-related distress compared to men [48,51,57]. This pattern was evident across different study designs and assessment methods, and it was apparent even before treatment initiation. While both genders experienced emotional strain, women seemed more susceptible to depressive and anxiety symptoms, while men generally reported lower overall levels of distress during this phase. Importantly, evidence suggested that psychological distress before IVF was more strongly associated with general psychological characteristics than with infertility-specific variables. Studies examining predictors of distress found that maladaptive coping strategies, self-criticism, dependency, and intrusive thoughts were key contributors to elevated psychological distress [33,48]. In comparison, adaptive coping strategies, such as seeking social support, were associated with lower distress levels [33]. Hence, pre-existing psychological profiles significantly influence emotional responses before IVF treatment starts.

#### 3.4.2. Gender Differences and Associations with Treatment Outcomes

Consistently, marked gender differences in emotional reactions were reported in 2 studies. Women experienced higher levels of anxiety and depressive symptoms compared to men and the general population, regardless of treatment outcomes [51,57]. Men, on the other hand, typically reported distress levels that were similar to or even lower than those of the general population, especially when their treatment was unsuccessful. Moreover, a strong emotional connection, particularly among men, was associated with higher treatment success rates. Thus, emotional involvement in the treatment process can have complex implications that differ based on gender [57].

#### 3.4.3. Psychological Trajectories Across Treatment Outcomes

One longitudinal study has shown that psychological responses change throughout the course of IVF treatment and vary significantly depending on the treatment outcome. To be more precise, Verhaak et al. [48] found that women who underwent unsuccessful IVF cycles experienced significant increases in anxiety and depressive symptoms after completing treatment, highlighting the emotional toll of treatment failure and unmet expectations. In contrast, women who achieved pregnancy reported a reduction in anxiety and depression levels during the same period, indicating a relief associated with successful outcomes. Despite differing outcomes, follow-up assessments showed that a significant number of women continued to experience subclinical levels of anxiety and/or depression several months after completing treatment, regardless of the treatment results. This finding emphasizes that psychological distress can persist beyond the immediate treatment phase and highlights the long-term emotional effects of infertility and IVF treatment, even after clinical resolution.

#### 3.4.4. Psychosocial Factors and Treatment Discontinuation

Only one study examined treatment continuation and discontinuation, identifying psychosocial factors as essential determinants of IVF treatment decisions. This study, conducted by Van Dongen et al. [47], involved a large cohort of couples evaluated before starting IVF treatment. It found that a minority of couples discontinued IVF within one year for personal reasons. This decision was significantly linked to a longer duration of infertility, lower perceived social support among women, and higher levels of infertility acceptance in both partners. The authors of this study concluded that both relational and individual psychosocial factors influence the decision to stop treatment, beyond purely medical considerations. Although depression and anxiety scores did not show statistically significant differences between men and women before and after IVF, women consistently reported higher levels of emotional distress across assessments, indicating a sustained psychological burden throughout the treatment process [47].

### 3.5. The Psychological Impact of IVF During Different Stages of IVF Cycle

A total of 25 studies examined the psychological impact of IVF or ART across different stages of the treatment cycle, including ovarian stimulation, oocyte retrieval, embryo transfer, and the waiting period before pregnancy testing. Despite heterogeneity in study design and assessment tools, consistent patterns emerged regarding anxiety, depression, quality of life, and gender differences during active treatment phases. Detailed study characteristics and quantitative outcomes are presented in Table 3.

#### 3.5.1. Gender Differences in Anxiety, Depression, and Infertility-Related Stress Across IVF Stages

Across the IVF cycle, psychological distress varied and generally increased as treatment progressed, with higher levels observed during active treatment phases and periods of uncertainty [18,40,46]. Although both women and men experienced psychological burden, women consistently demonstrated greater vulnerability to emotional distress, particularly in relation to treatment expectations, infertility-related concerns, and fear of negative outcomes [19,28,41,50]. Interestingly, Chiaffarino et al. [40] showed that anxiety and depressive symptoms increased during active IVF treatment phases, particularly around cycle suspension or pregnancy testing, with women exhibiting higher prevalence and incidence of distress than men, while Ismail et al. [46] identified the period before pregnancy testing as the most psychologically vulnerable stage for both partners.

Additionally, women consistently reported higher levels of anxiety, depressive symptoms, and infertility-related stress than men at most stages of the IVF cycle, a finding replicated across diverse populations and measurement instruments [19,28,34,37,39,41,53,54,56]. Men generally reported lower overall distress [18,19,27,28,34,36,62]; however, they still experienced elevated stress during critical treatment phases, particularly in relation to financial pressure, treatment uncertainty, and partner distress [28,37,40,46,61]. Despite these differences in anxiety levels, the prevalence of diagnosed anxiety disorders was comparable between genders in one study, with 4.8% of women and 4.9% of men meeting the criteria based on PRIME-MD [62]. These findings indicate that while women tend to report higher anxiety, depression and infertility-related stress levels, both genders experience psychological distress related to infertility, highlighting the need for targeted mental health support.

#### 3.5.2. Gender Differences in Quality of Life During Active IVF Treatment

Research evaluating quality of life during IVF has consistently shown that women report lower health-related and fertility-specific quality-of-life scores compared to men throughout the treatment process [18,27,31,36]. For instance, in a longitudinal observational study [20] evaluating quality of life at three points: at the beginning of ovarian stimulation, during oocyte retrieval before discharge, and approximately 14 days after embryo transfer, a significant gender effect was observed across all dimensions of the SF-36 questionnaire, with women have lower mean scores overall than men [18]. To be more precise women reported experiencing poorer emotional, social, and vitality-related functioning [18], while other studies revealed a statistically significant decline in their quality of life following repeated IVF failures [27,36]. In contrast, men generally maintained higher quality-of-life scores, although declines were noted in cases of prolonged treatment or increased infertility-related stress [18,28,41].

### 3.6. Psychological Reactions for Those Conceived with IVF

A total of 5 studies investigated the psychological impact of IVF in women and men who conceived with IVF. The results of these studies are represented in Table 4.

#### 3.6.1. Infertility-Related Stress and Pregnancy-Related Anxiety Following IVF Conception Between Couples

Across three studies, pregnancy-related anxiety emerged as the most prominent psychological response among women and men who conceived through IVF [32,43,44]. A comparative study [44] evaluated anxiety levels, emotional responses to pregnancy, relationship adjustment, and reactions to recalled infertility experiences at 13 weeks of gestation in individuals who conceived through IVF compared with those who conceived naturally. This study showed that both women and men in the IVF group reported higher anxiety related to pregnancy loss than their naturally conceiving counterparts. Women who conceived through IVF exhibited greater muscle tension and heightened anxiety regarding pregnancy loss, while men reported elevated levels of physical worry, indirect aggression, guilt, and emotional detachment. Notably, men experiencing high levels of infertility-related distress demonstrated increased anxiety about the baby’s health [46]. Similarly, in a longitudinal study [43], emotional reactions and pregnancy experiences were assessed at multiple gestational time points in couples who conceived through IVF compared to those who conceived naturally. The results indicated that overall concerns about pregnancy loss were higher among IVF couples than in the control group. Women in the IVF group exhibited greater anxiety early in pregnancy; however, this anxiety significantly decreased as the pregnancy progressed. They also reported a more positive pregnancy experience, lower anxiety regarding the baby’s gender, and fewer concerns about losing personal freedom as parents. In contrast, IVF men demonstrated heightened anxiety related to the potential for injury to the baby during childbirth. Their anxiety about the baby’s health increased between weeks 13 and 26 of gestation, a pattern not observed in men who conceived naturally. Further, women who recalled higher levels of distress related to previous childlessness reported greater anxiety about pregnancy loss and the baby’s health [43].

#### 3.6.2. Depressive Symptoms After Successful IVF

In contrast to anxiety-related outcomes, depressive symptoms experienced after a successful IVF conception are generally similar to those found in individuals who conceive naturally. Two studies have shown no significant differences in the prevalence or severity of depressive symptoms between women and men who conceived through IVF and those who conceived naturally during pregnancy and the first year of parenthood [55,60]. These findings suggest that successful conception via IVF does not seem to be associated with an increased risk of depression during the post-conception period.

#### 3.6.3. Couple Functioning and Relational Adjustment

Studies that examined relational and psychosocial adjustment following IVF conception found no evidence of impaired couple functioning. The quality of relationships and marital adjustment among IVF couples is similar to that of couples who conceive naturally. In fact, some studies suggest that the shared experience of infertility and the stress associated with treatment may help to strengthen partner relationships during the transition to parenthood [55,60]. In addition, emotional bonding, partner support, and parental self-efficacy were generally maintained following successful IVF.

### 3.7. COVID-19 Related IVF Psychological Reactions

Only one study investigated the psychological effects of interruptions or postponements of IVF treatment due to COVID-19 among infertile couples [22]. The findings indicated that disruptions in therapy during the pandemic were linked to increased psychological distress. Women consistently reported higher levels of anxiety and depressive symptoms compared to men. Also, women over the age of 35 and those with previous IVF attempts were found to be particularly vulnerable to heightened emotional distress. Both situational and individual factors contributed to psychological distress. Increased exposure to COVID-19-related news and having a partner with a pre-existing psychological disorder were linked to higher levels of anxiety and depression. Further, women with underlying reproductive conditions, such as poor ovarian reserve, endometriosis, or uterine fibroids, demonstrated greater psychological vulnerability during interruptions in treatment. The study concluded that the sudden disruption of fertility treatment during the pandemic had a disproportionate impact on women and individuals with existing medical or psychosocial risk factors, underlining the need for targeted psychological support during times of treatment uncertainty (Table 5).

### 3.8. Long Term IVF Psychological Reactions

A total of 4 studies investigated the long-term psychological impact of IVF in women and men. The results of these studies are represented in Table 6.

#### 3.8.1. Long-Term Psychological Distress and Gender-Specific Experiences

Long-term psychological outcomes following IVF were strongly influenced by treatment outcome, continuation of fertility treatment, and parenthood status. Longitudinal interview-based research indicated that anxiety and depressive symptoms fluctuated over time but tended to return to baseline following successful treatment outcomes, emphasizing the role of infertility resolution in long-term psychological adjustment [25]. Evidence from a long-term follow-up study of couples with unsuccessful IVF outcomes showed persistent psychological challenges several years after treatment, with clear gender-specific patterns [42]. Women more frequently reported problems related to self-image, psychological distress, and loss of hope, often accompanied by difficulties in marital relationships. Men, in contrast, primarily reported psychological issues and experiences related to adoption. Thus, infertility seems to impact emotional well-being and identity in distinct ways for women and men, long after treatment has concluded.

#### 3.8.2. Treatment Continuation and Discontinuation on Long-Term Psychological Impact

It is worth noting that long-term psychological outcomes differed between couples who pursued further fertility treatment after IVF failure and those who discontinued treatment. Couples who did not continue with further treatment appeared to be more adversely affected in the long term, with both women and men showing stronger associations with psychological distress and the recognition of the impossibility of conceiving a child [42]. Conversely, couples who continued treatment or pursued alternative family-building options, such as adoption, demonstrated comparatively better long-term psychological adjustment. The decision to abandon treatment was strongly associated with recognizing infertility as permanent, underscoring the emotional burden linked to treatment discontinuation.

#### 3.8.3. Role of Parenthood Status and Treatment Success

Cross-sectional studies with extended follow-up demonstrated that long-term psychological well-being after IVF is strongly influenced by treatment success and parenthood status [9,64]. Parents, whether biological or adoptive, reported better psychological adjustment, including higher quality of life, a stronger sense of coherence, and greater positive well-being, several years after treatment, compared to those who remained childless. In contrast, individuals with unsuccessful IVF outcomes exhibited a lower sense of coherence and higher levels of depressive symptoms and psychological distress, regardless of gender, indicating that persistent childlessness is associated with long-term emotional vulnerability [9,64].

Johansson et al. [64] further showed that individuals without children experienced significantly poorer psychological outcomes, particularly in terms of depression and reduced positive well-being, whereas those living with children demonstrated more favorable long-term mental health profiles. Similarly, comparative evidence from Johansson et al. [9] indicated that both women and men in the unsuccessful IVF group reported lower quality of life and greater psychological distress than their counterparts with successful IVF outcomes. Research suggests that among individuals who become parents through IVF, men tend to report a higher quality of life, fewer depressive symptoms, and greater self-confidence compared to women. This implies that men’s long-term psychological well-being may be especially affected by the resolution of infertility.

### 3.9. NOS- Risk of Bias Assessment Results

According to the NOS, scores ranged from 2 to 8 stars. One study had an overall score of 2 stars [38], 1 study scored 3 stars [63], 7 studies scored 5 stars [25,26,42,46,52,56,59], 9 studies scored 6 stars [9,18,21,24,39,47,51,53,64], 26 studies scored 7 stars [19,20,22,28,29,30,31,32,33,34,35,36,37,41,43,44,45,48,49,54,55,57,60,61,62], 3 studies scored 8 stars [23,27,40]. Regarding the selection domain, 13 studies reported the response rate of infertile couples or patients [9,27,36,41,45,47,50,51,54,55,60,61], while 20 studies included sample sizes of 400 participants or more [9,19,20,21,23,27,29,30,35,38,40,41,47,50,54,58,60,61,62,64]. With respect to the comparability domain, 13 studies did not adequately control for potential confounders through subgroup or multivariable analyses [9,21,25,26,38,42,46,51,52,53,56,59,63]. Lastly, for the outcome domain, one study relied exclusively on self-reported data without reference to original medical records [63], and one study did not sufficiently describe the statistical analysis methods used [56] (Table 6).

**Table 6:** Newcastle-Ottawa Scale for risk of bias assessment of the included studies.

	NOS Quality Assessment Criteria				
Authors	Selection	Comparability	Outcome	Total NOS Score	Risk of Bias
Agostini et al. [18]	⚝⚝	⚝	⚝⚝⚝	6	Moderate
Bai et al. [19]	⚝⚝⚝	⚝	⚝⚝⚝	7	Low
Barra et al. [22]	⚝⚝⚝	⚝	⚝⚝⚝	7	Low
Benyamini et al. [39]	⚝⚝	⚝	⚝⚝⚝	6	Moderate
Boivin & Schmidt [54]	⚝⚝⚝	⚝	⚝⚝⚝	7	Low
Chiaffarino et al. [40]	⚝⚝⚝⚝	⚝	⚝⚝⚝	8	Low
Courbiere et al. [21]	⚝⚝⚝	-	⚝⚝⚝	6	Moderate
Cusatis et al. [22]	⚝⚝⚝	⚝	⚝⚝⚝	7	Low
Donarelli et al. [41]	⚝⚝⚝	⚝	⚝⚝⚝	7	Low
Fekkes et al. [23]	⚝⚝⚝⚝	⚝	⚝⚝⚝	8	Low
Filetto & Makuch [42]	⚝⚝	-	⚝⚝⚝	5	Moderate
Franco et al. [63]	⚝⚝	-	⚝	3	High
Gelgoot et al. [35]	⚝⚝⚝	⚝	⚝⚝⚝	7	Low
Gibson et al. [55]	⚝⚝⚝	⚝	⚝⚝⚝	7	Low
Gonen & Bokek-Cohen [24]	⚝⚝	⚝	⚝⚝⚝	6	Moderate
Hjelmstedt et al. [44]	⚝⚝⚝	⚝	⚝⚝⚝	7	Low
Hjelmstedt et al. [43]	⚝⚝⚝	⚝	⚝⚝⚝	7	Low
Holter et al. [45]	⚝⚝⚝	⚝	⚝⚝⚝	7	Low
Ismail et al. [46]	⚝⚝	-	⚝⚝⚝	5	Moderate
Järvholm et al. [25]	⚝⚝	-	⚝⚝⚝	5	Moderate
Johansson et al. [64]	⚝⚝	⚝	⚝⚝⚝	6	Moderate
Johansson et al. [9]	⚝⚝⚝	-	⚝⚝⚝	6	Moderate
Kondaveeti et al. [26]	⚝⚝	-	⚝⚝⚝	5	Moderate
Lopes et al. [36]	⚝⚝⚝	⚝	⚝⚝⚝	7	Low
McNaughton-Cassill et al. [56]	⚝⚝⚝	-	⚝⚝	5	Moderate
Merari et al. [57]	⚝⚝⚝	⚝	⚝⚝⚝	7	Low
Onat & Aba [28]	⚝⚝⚝	⚝	⚝⚝⚝	7	Low
Peterson et al. [29]	⚝⚝⚝	⚝	⚝⚝⚝	7	Low
Peterson et al. [30]	⚝⚝⚝	⚝	⚝⚝⚝	7	Low
Phromyothi et al. [53]	⚝⚝⚝	-	⚝⚝⚝	6	Moderate
Pottinger et al. [59]	⚝⚝	-	⚝⚝⚝	5	Moderate
Ragni et al. [58]	⚝⚝⚝	⚝	⚝⚝⚝	7	Low
Rashidi et al. [31]	⚝⚝⚝	⚝	⚝⚝⚝	7	Low
Repokari et al. [60]	⚝⚝⚝	⚝	⚝⚝⚝	7	Low
Samorinha et al. [61]	⚝⚝⚝	⚝	⚝⚝⚝	7	Low
Schaller et al. [37]	⚝⚝⚝	⚝	⚝⚝⚝	7	Low
Stevenson et al. [32]	⚝⚝⚝	⚝	⚝⚝⚝	7	Low
Tome & Zwahlen [38]	⚝⚝	-	-	2	High
Van den Broeck et al. [33]	⚝⚝⚝	⚝	⚝⚝⚝	7	Low
Van Dongen et al. [47]	⚝⚝	⚝	⚝⚝⚝	6	Moderate
Madero et al. [27]	⚝⚝⚝⚝	⚝	⚝⚝⚝	8	Low
Verhaak et al. [48]	⚝⚝⚝	⚝	⚝⚝⚝	7	Low
Volgsten et al. [62]	⚝⚝⚝	⚝	⚝⚝⚝	7	Low
Volgsten et al. [49]	⚝⚝⚝	⚝	⚝⚝⚝	7	Low
Wichman et al. [51]	⚝⚝⚝	-	⚝⚝⚝	6	Moderate
Winter et al. [34]	⚝⚝⚝	⚝	⚝⚝⚝	7	Low
Yassini et al. [52]	⚝⚝	-	⚝⚝⚝	5	Moderate

Note: The original NOS was applied to cohort studies, and a modified NOS version was used for cross-sectional studies. Stars indicate the number of criteria fulfilled within each NOS domain, with higher numbers of stars reflecting better methodological quality in the selection, comparability, and outcome (or exposure) assessment domains. Studies scoring 7–9 points were classified as low risk of bias, those scoring 4–6 points as moderate risk, and those scoring 0–3 points as high risk of bias according to the NOS. NOS: Newcastle–Ottawa Scale.

## 4. Discussion

Our study revealed that IVF can be a significant source of psychological distress for both women and men. However, women consistently exhibit higher levels of depression and anxiety across all stages of IVF treatment. Psychological distress may emerge even before treatment initiation, persist throughout the treatment process, and continue in the long term, particularly following unsuccessful IVF attempts. The emotional burden associated with IVF appears to be multifactorial, reflecting uncertainty regarding treatment outcomes, physical and hormonal demands, financial strain, and societal or personal expectations surrounding parenthood [65]. These findings underscore the importance of integrating psychological support for individuals and couples undergoing IVF across all stages of the treatment trajectory to mitigate adverse mental health outcomes.

Interpretation of these findings must consider the substantial methodological heterogeneity among the included studies. Most investigations employed observational designs, predominantly cross-sectional, with fewer longitudinal studies, limiting causal inference regarding psychological outcomes across IVF stages. In addition, considerable variability was observed in the timing of psychological assessments, ranging from pre-treatment planning and active treatment phases to pregnancy and long-term follow-up. However, only one study was conducted during the COVID-19 pandemic, limiting the generalizability of findings on treatment interruption and pandemic-related psychological stress. Except from differences in study design and timing of assessment, comparability across studies is further constrained by substantial heterogeneity in the psychological instruments used to assess distress, anxiety, depression, and quality of life. As illustrated in Table 1, studies employed a wide range of self-report measures, including general psychiatric screening tools such as the Beck Depression Inventory (BDI), Patient Health Questionnaire-2 (PHQ-2), Hopkins Symptom Checklist (HSCL), and PROMIS Anxiety, alongside infertility-specific instruments such as the Fertility Problem Inventory (FPI) and Fertility Quality of Life (FertiQoL). While these tools are all validated, they capture partially overlapping but conceptually distinct dimensions of psychological functioning, ranging from general affective symptoms to infertility-related stress, coping, and relational impact. Indeed, several studies relied on brief screening measures with different sensitivity thresholds (e.g., PHQ-2 versus BDI). In contrast, others used multidimensional instruments without standardized clinical cut-off points, limiting direct comparison of prevalence estimates across samples.

Variability in research findings also arises from differences in scoring methods and the interpretative thresholds used to define clinically meaningful distress. Some studies report continuous scores and analyze gender differences in mean values, while others categorize participants based on specific cut-off points for mild, moderate, or severe symptomatology, often using criteria that are unique to the scale or the sample being studied. In addition, the timing of assessments relative to the IVF process varies even within the same treatment stage. Psychological outcomes may be measured during initial planning, pre-treatment counseling, or just before the start of a cycle, thereby capturing different anticipatory stress responses. These methodological inconsistencies likely contribute to the variability observed in effect sizes and prevalence rates across studies, even though the overall trend of a higher psychological burden in women remains consistent.

Moreover, the assessment of psychological distress in populations undergoing IVF should consider the potential impact of surveillance bias. Individuals and couples undergoing IVF typically experience frequent clinical visits, repeated medical evaluations, and close interactions with healthcare professionals. This level of monitoring may lead to a higher likelihood of detecting symptoms of anxiety or depression compared to the general population. As a result, the increased observation could contribute to a higher reported prevalence of psychological symptoms, especially when using self-report screening tools. Consequently, some differences observed between IVF patients and non-infertile populations may reflect increased detection rather than an actual rise in underlying psychological issues. However, the consistent presence of gender differences and stage-specific patterns of distress across studies suggests that surveillance bias alone is unlikely to explain the findings fully. Future research would benefit from study designs that include appropriately matched control groups, similar assessment frequencies, or analytical strategies that account for varying levels of healthcare exposure. Despite these restrictions, consistent patterns emerged across diverse methodologies, particularly with respect to gender-related differences in psychological distress and stage-specific vulnerability, supporting the overall robustness of the review’s conclusions while underscoring the need for cautious interpretation.

Research shows that women experience higher stress levels than men when seeking specialized infertility care. Over time, reported stress increased in both genders, rising from 22% in 2003 to 40% in 2009 among women and from 11% to 31% among men. At the same time, the use of stress management strategies declined markedly, particularly among women (from 98% in 2003 to 58% in 2009), with a similar decline among men (from 98% to 29%) [26]. In terms of health-related quality of life, men consistently scored higher across all SF-36 domains, with significant gender differences in social functioning (83.7 vs. 77.6, *p* < 0.0001), role-emotional functioning (87.6 vs. 80.3, *p* < 0.0001), and mental health (74.7 vs. 69.2, *p* < 0.0001) [58]. Women exhibit higher anxiety (PSS-4: 6.30 vs. 5.26, *p* < 0.001) and depressive symptomatology (BDI: 6.1 vs. 4.0, PHQ-2: 1.47 vs. 1.10, *p* = 0.002) [30,35], with a mean concern score of 2.43 (SD = 1.19) compared to 2.26 (SD = 1.26) for men [35]. Women reported significantly higher levels of stress across all subscales of the FPI compared to men (*p* < 0.01). The most notable differences highlight how gender affects coping mechanisms in several areas: social anxiety (27.1 ± 11.4 for women vs. 22.4 ± 9.1 for men), sexual anxiety (16.7 ± 7.6 vs. 13.9 ± 5.5), relationship anxiety (20.0 ± 9.0 vs. 18.6 ± 7.6), the need for fertility (36.8 ± 11.0 vs. 32.6 ± 10.2), and general anxiety (128.9 ± 35.2 vs. 114.5 ± 28.3) [29]. Despite men showing improved understanding of infertility (84% in 2009 vs. 67% in 2003), women’s scores have remained stable (82% in 2009 vs. 84% in 2003) [26]. These findings emphasize the need for targeted psychological interventions that account for gender disparities, ensuring tailored support to help both men and women cope effectively with the emotional burden of infertility and IVF treatment. The results of this study are consistent with a systematic review by Ying et al. [66], which indicated that women undergoing IVF reported higher levels of anxiety and depression compared to fertile women. Moreover, Verhaak et al. [4], in a relevant analysis, noticed that while the emotional distress experienced by women at the beginning of an IVF treatment was more pronounced, the difference between them and fertile women was relatively small. This suggests that while IVF presents a significant psychological burden, the emotional toll, though notable, does not create an overwhelming gap in distress levels between these groups.

Analysis of studies on the psychological impact of IVF treatment reveals significant gender differences among individuals dealing with infertility, whether evaluated immediately before starting treatment or at the beginning, with or without post-IVF cycle assessments. Specifically, during the initial phase of IVF treatment, both men and women experience psychological distress, but the results are inconclusive. Women were found to experience higher levels of depression (BDI: 4.0 vs. 2.7, *p* < 0.001) and anxiety (STAI: 32.8 vs. 30.4, *p* = 0.002), compared to men [51]. Likewise, women’s psychological reactions vary significantly depending on whether the IVF cycle is successful or unsuccessful; depression and anxiety levels were also higher in women, regardless their success in conceiving. However, husbands of women who successfully conceived reported higher depression scores than those whose partners did not conceive. In contrast, non-conceiving husbands had depression and trait anxiety scores that were notably lower than the normative level [57]. Furthermore, considering pre- and post-IVF psychological evaluation, while most women adapt well to unsuccessful IVF treatments, a significant proportion continue to experience emotional distress months later. Over 20% reported subclinical anxiety and depression six months after their last treatment. Women showed a statistically significant increase in anxiety (t (1,64) = −2.5, *p* = 0.02) and depression (t (1,64) = −2.9, *p* = 0.01) between pre-treatment and post-treatment (T1 to T2). Key factors influencing emotional adaptation included personality traits, the perceived significance of fertility issues, and social support. In contrast, men showed no significant changes in anxiety or depression across all time points, suggesting greater emotional stability following unsuccessful treatment [4]. However, even though Dongen et al. [47] indicated that there were no statistically significant differences in psychological distress levels between men and women before and after IVF treatment, women tend to experience higher levels of anxiety, depression, and overall psychological distress compared to men at both points in time.

Our systematic review also revealed that multiple studies have consistently demonstrated significant gender differences in psychological distress during infertility treatment. Women exhibited lower quality of life scores across all SF-36 dimensions, with significant differences in emotional, general health, vitality, and social function indices (*p* < 0.001) [18]. Similarly, FertiQoL assessments confirmed lower well-being in women, particularly in emotional, mind–body, and social dimensions [27]. Depression levels were also higher among women undergoing IVF, with 60.8% experiencing mild to severe symptoms compared to 53.1% of men [52]. Anxiety levels followed a similar pattern, with women consistently scoring higher on STAI assessments than men (*p* < 0.01) [28,37,61]. Significant predictors of depression differed by gender, with social and sexual anxiety playing a larger role for women, while men were more affected by treatment costs and social anxiety. Additionally, attachment styles were found to influence infertility stress, with cross-partner effects highlighting the impact of one partner’s distress on the other [41]. Despite these differences, both men and women experienced psychological distress, underscoring the need for gender-specific mental health support during infertility treatment. Similarly to our results, Ying et al. [66] observed that infertile women experience higher levels of depression and anxiety during key stages of IVF treatment, including oocyte retrieval, embryo transfer, and before the pregnancy test, compared to the pretreatment period. Men, on the other hand, reported elevated depression only during the waiting period for IVF results, with anxiety levels remaining relatively unchanged throughout the cycle. Gender differences were evident, with women generally experiencing higher levels of anxiety and depression, while men exhibited more stable anxiety levels but higher depression, potentially influenced by societal expectations for emotional restraint. Other studies have shown that women also faced physical discomfort from the procedures, particularly oocyte retrieval, and emotional distress due to concerns over embryo quality and self-image. The waiting period before the pregnancy test was particularly stressful for both genders, as couples felt powerless and uncertain about the outcome [67,68]. Meanwhile, in a recent systematic review, Zanettoullis et al. [69] concluded that both chronic and acute stress significantly impact the egg retrieval stage, with failure at this time point being positively correlated with elevated anxiety scores and biomarkers. Chronic stress was predominantly associated with fertilization and embryo transfer stages, as well as with lower pregnancy rates. These findings highlight the crucial role of stress in the IVF process, particularly during key treatment milestones. However, in this systematic review, the influence of gender as a confounding factor was not evaluated [69].

Moreover, our systematic review revealed that research on couples who conceive through IVF has shown that while they adjust to parenthood similarly to naturally conceiving couples, there are subtle differences, particularly in their concerns and confidence regarding parenthood. IVF couples, particularly women, experience higher levels of anxiety, especially concerning pregnancy loss, but report more positive pregnancy experiences compared to those in the group who conceived naturally. Men in the IVF group tend to be more concerned about the baby’s health and show an increase in anxiety levels as the birth date approaches. However, anxiety levels in IVF women decrease as pregnancy progresses, a pattern not observed in men, who experience an increase in anxiety over time. Interestingly, marital satisfaction and dyadic cohesion are largely unaffected by IVF, with no significant differences between ART couples and the group who conceived naturally. However, depressive symptoms during pregnancy negatively impacted relationship dynamics only in control couples, suggesting greater psychological resilience in ART couples. IVF couples also exhibit different personality traits and emotional responses to pregnancy, which may require additional support, particularly for women, to manage the psychological and emotional stress of IVF treatment [32,43,44,55,60].

Additionally, considering the long-term effects of IVF treatment, our study noticed that both men and women remain psychologically affected three years after undergoing pre-implantation genetic diagnosis (PGD). Both genders expressed concerns that their relationships had been impacted, both positively and negatively, with ongoing feelings of anxiety and depression. Women undergoing standard IVF experienced significantly higher levels of anxiety compared to men, with their psychological symptoms fluctuating throughout the process. While these levels generally returned to baseline following successful PGD, women still faced a greater psychological burden than men. Men, although affected, primarily expressed concerns about their partner’s status, whereas women were more likely to report direct anxiety and depression related to the IVF and PGD process. These findings suggest that while PGD offers some psychological relief, the emotional impact of infertility treatments is more persistent and intense for women [25]. Results also revealed that despite IVF treatment failure, over 75% of couples were living with children 4 to 5.5 years later, and those with children reported improved quality of life. However, women still faced greater psychological challenges, highlighting the gendered differences in emotional and psychological well-being related to infertility [9]. Additionally, after 3–8 years from the last IVF cycle, women exhibited a stronger emotional connection to issues related to self-image, psychological distress, and feelings of hopelessness, particularly when considering adoption, whereas men were more focused on the psychological difficulties of adoption itself. These findings emphasize the significant psychological burden of infertility treatment, with 82.6% of couples not pursuing further treatment, suggesting a recognition of the emotional toll and potential abandonment of further fertility treatments [42]. In agreement to our results, another systematic review showed that women who had a successful IVF cycle reported lower negative emotions compared to pretreatment levels [68]. Likewise, a long-term study by Bryson et al. [8] revealed that women who remained childless 4 to 9 years after unsuccessful IVF treatment reported lower life satisfaction than those who eventually became parents. These results suggest that the unsuccessful outcome of IVF, rather than the IVF process itself, has long-term psychological consequences.

Moreover, our systematic review identified a study that examined the psychological impact of interrupted or postponed IVF treatments due to the COVID-19 pandemic. The findings revealed that the pandemic significantly exacerbated psychological distress in infertile couples, resulting in elevated levels of anxiety and depression. Furthermore, the delay in scheduled IVF treatments contributed to increased psychological burden for both men and women, with heightened anxiety and depressive symptoms observed across genders [20].

The observed gender differences and stage-specific patterns of psychological distress have significant implications for clinical practice in IVF settings. Routine psychological screening for anxiety, depression, and stress related to infertility should be integrated into standard fertility care for both women and men, starting at the pre-treatment stage and continuing throughout the IVF process. Women may benefit from early and repeated screenings due to their consistently higher vulnerability to emotional distress. However, men should not be overlooked, especially during critical phases such as treatment failure, prolonged infertility, or financial and decision-related stress. Targeted psychosocial interventions, including counseling, stress management programs, and couple-based support, can help address maladaptive coping strategies, attachment-related distress, and relationship strain. Incorporating mental health professionals within multidisciplinary fertility teams can facilitate the timely identification of at-risk individuals and support emotional well-being, treatment adherence, and overall quality of life during and after IVF treatment.

Lastly, this systematic review has both strengths and limitations. Τhis systematic review explored gender differences in the psychological impact of IVF, emphasizing how the treatment contributes to increased anxiety, depression, and a decline in various aspects of quality of life. The findings highlight the emotional distress and unfavorable circumstances faced by couples, which exacerbate psychological challenges for both men and women during the IVF process. However, women were found to experience higher levels of psychological distress, including increased anxiety and depression symptoms, compared to men at nearly all stages of IVF treatment.

Nevertheless, several limitations warrant acknowledgment. Most included studies employed observational designs, predominantly cross-sectional, which restrict causal inference and limit conclusions regarding temporal changes in psychological distress across IVF stages. However, although many included studies achieved moderate to high scores on the NOS, most of the studies classified as having low risk of bias were nonetheless cross-sectional in design, reflecting acceptable methodological quality within inherent design constraints. A substantial proportion of studies were rated as having moderate risk of bias, relied on cross-sectional designs, which inherently limit causal inference, and were often accompanied by incomplete adjustment for potential confounding variables. Thus, while the overall evidence base reveals consistent patterns in psychological distress and gender-related differences during IVF, the findings should be interpreted with appropriate caution.

Another important limitation relates to the conceptual distinction between sex and gender. Although the title and scope of this review refer to gender differences, the majority of included studies examined psychological outcomes based on binary biological sex (women and men), as reported in clinical and demographic data. Few studies explicitly addressed socio-cultural aspects of gender, such as gender roles, identity, or norms, which may also influence psychological responses to infertility and IVF treatment. Consequently, the findings primarily reflect sex-based differences rather than a comprehensive gender-based analysis, and caution is warranted when interpreting results in a broader gender framework. In addition, considerable heterogeneity was observed in psychological assessment tools, outcome definitions, and measurement timing, complicating direct comparisons across studies and potentially contributing to variability in reported effect sizes and prevalence estimates. However, even though efforts were made to conduct a comprehensive literature search, the inclusion of only English-language publications may have introduced language-related publication bias. Furthermore, excluding randomized controlled trials, even when aligned with examining natural psychological responses rather than intervention effects, limits insight into the efficacy of specific psychosocial support strategies. Finally, most studies were conducted in high-income countries and before the COVID-19 pandemic, with only one study capturing pandemic-related disruptions, potentially limiting the generalizability of findings to other healthcare contexts or periods of systemic stress. These limitations highlight the need for more standardized, longitudinal, and culturally diverse research to further clarify gender-specific psychological trajectories in IVF.

## 5. Conclusions

In conclusion, this systematic review demonstrates that infertility and IVF are associated with substantial psychological burden for both women and men across multiple stages of the treatment trajectory. Psychological distress can emerge before treatment initiation, intensify during active IVF procedures, and persist in the long term, particularly following unsuccessful treatment outcomes. Across studies, women consistently reported higher levels of anxiety, depressive symptoms, infertility-related stress, and reductions in quality of life compared with men. However, men also experienced meaningful psychological strain, especially during critical treatment phases and in response to treatment failure. The findings highlight clear gender-specific patterns in emotional responses to infertility and IVF, suggesting that psychological distress is shaped not only by treatment stage but also by relational dynamics, coping strategies, and expectations related to parenthood. Significantly, psychological responses extended beyond medical aspects of treatment to social functioning, emotional well-being, and couple relationships, underscoring the multidimensional impact of IVF.

To sum up, there is urgent need for a gender-sensitive and stage-specific approach to psychological care in fertility settings. Integrating routine psychological assessment and tailored psychosocial support throughout the IVF process may improve emotional well-being, support treatment adherence, and enhance overall quality of life for individuals and couples undergoing fertility treatment.

## Figures and Tables

**Table 1:** Studies with psychological reactions during the IVF or ART planning period.

Authors, Year	Design	Country	Sample Size	Psychological Outcomes Assessed	Materials	Main Results
Cusatis et al. [22], 2019	Cross-sectional study	USA	156 participants (90 women, 66 men)	Quality of life, anxiety	FertiQoL tool and PROMIS Anxiety instrument.	Women reported lower fertility-related quality of life and higher anxiety than men; increased reflective thinking was associated with greater stress in both genders
Kondaveeti et al. [26], 2011	Cross-sectional study	Ireland	180 couples	Anxiety, depression, psychosocial functioning	Psychosocial questionnaire that included questions about family support, relationships, understanding of infertility, and stress management was given two times (2003 vs. 2009).	Women showed higher emotional distress than men; infertility-related stress affected multiple psychosocial domains
Peterson et al. [29]	Cross-sectional study	Canada	1026 individuals (520 women, 506 men)	Stress, coping, psychosocial adjustment	WCQ, FPI and DAS	Increased stress levels over time and reduced use of stress management strategies, particularly among women
Peterson et al. [30]	Comparative cross-sectional study	USA	1026 individuals (520 women, 506 men)	Depression	WCQ BDI	Higher depressive symptoms have been observed, with women demonstrating a higher level of vulnerability.
Ragni et al. [58]	Cross-sectional study	Italy	1000 couples	Quality of life	SF-36 short form	SF-36 scores for men were higher than those for women. Significant differences were observed in HRQoL based on duration of infertility and previous IVF attempts.
Gelgoot et al. [35]	Cross-sectional study	Canada	313 female and 254 male patients	Anxiety, depression and concern score	PSS-4 PHQ-2 Infertility Concern Scale	Compared with men, women reported higher levels of concern and anxiety, whereas men exhibited lower levels of depressive symptoms.
Fekkes et al. [23]	Cross-sectional study	Netherlands	425 men and 447 women	Health-related quality of life	HSCL, SIP, IBI and IPC scales	Both women and men aged 21–30 years showed significantly poorer social and emotional functioning than the general population, with younger women experiencing more pronounced difficulties.

Note: FertiQoL: Fertility Quality of Life; PROMIS: Patient-Reported Outcomes Measurement Information System; WCQ: Ways of Coping Questionnaire; DAS: Dyadic Adjustment Scale; FPI: Fertility Problem Inventory; BDI: Beck Depression Inventory; SF-36: Short Form 36; RE: Role emotional; GE: general health; VT: Vitality; MH: Mental Health; BP: physical pain; RP: physical role; PF: physical functioning; SF: social function; PSS: Perceived Stress Scale; PHQ-2: Patient Health Questionnaire-2; HSCL: Hopkins Symptom Checklist; SIP: Sickness Impact Profile; ΙΒΙ: Irrational Beliefs Inventory; IPC: Irrational Parenthood Cognitions scale.

**Table 2:** Studies with Pre-IVF and or post-IVF psychological reactions.

Authors, Year	Design	Country	Sample Size	Psychological Outcomes Assessed	Materials	Main Results
Merari et al. [57], 2002	Cross-sectional study	Israil	113 childless couples who suffered from infertility of unknown or mechanical (women) cause	Depression, anxiety, emotional reactions and attitudes	- Personal Background Questionnaire - DACL - Spielberger’s STAI - Olson’s FACES	Marked gender differences were observed, with women reporting elevated anxiety and depression relative to population norms, men showing lower distress in the non-conceiving group, and higher emotional engagement being associated with treatment success.
Wichman et al. [51], 2011	Retrospective cohort study	USA	162 couples	Psychological distress, anxiety and depression	- BDI - STAI - State-Trait Anger Inventory - IES (adapted to infertility) - Perceived Stress Scale -Wilcoxon signed rank test	Women reported significantly higher levels of depressive symptoms, anxiety, infertility-related distress, and overall perceived stress than men during IVF preparation.
Van den Broeck et al. [33], 2010	Cross-sectional study	Belgium	106 women and 102 men	Psychological distress	DEQ ECR-R UCL IES BSI	Distress was associated with maladaptive coping and self-criticism, with social support acting as a protective factor.
Van Dongen et al. [47], 2015	Cohort study	Netherlands	667 couples were analyzed before and after IVF	Depression and anxiety	SCREENIVF	Treatment discontinuation was associated with longer infertility and lower female social support; women showed higher anxiety and depression than men across time points
Verhaak et al. [48], 2005	Longitudinal, prospective study	Netherlands	148 IVF patients and 71 partners	Anxiety, depression, coping and social support	STAI, BDI, Eysenck’s, Illness Cognitions Questionnaire, Cope, Personality Inventory (Life Orientation Test) and questions about social support	Women showed significant increases in anxiety and depression after unsuccessful IVF with no recovery at 6-month follow-up, whereas men showed no significant changes across time; over 20% of women exhibited persistent subclinical distress.

Note: SD: Standard deviation; IVF: In vitro fertilization; GHQ-28: General Health Questionnaire; T1, T2 and T3: First, second and third assessment; ECR: Experiences in Close Relationships Scale; STAI: State Trait Anxiety Inventory; BDI: Beck Depression Inventory; DEQ: Depressive Experiences Questionnaire; ECR-R: Experiences in Close Relationships-Revised; UCL: Utrechtse Coping List; IES: Impact of Event Scale; BSI: Brief Symptom Inventory; ICSI: Intracytoplasmic Sperm Injection; DACL: Lubin’s Depression Adjective Checklist; FACES: Family Adaptability and Cohesion Evaluation Scales.

**Table 3:** Studies with psychological reactions during the IVF cycles.

Authors, Year	Design	Country	Sample Size	Psychological Outcomes Assessed	Materials	Main Results
Agostini et al. [18], 2017	Longitudinal observational study	Italy	85 subfertile women and men undergoing ART treatment	QoL	Italian version of SF-36	Significant declines in emotional, general health, vitality, and social functioning were observed across IVF stages, regardless of clinical or demographic factors. Infertile women have a worse QoL throughout all phases of ARTs compared to men
Bai et al. [19], 2019	Cross-sectional study	China	380 women and 360 men undergoing infertility treatment	Depression and anxiety	PHQ-9 and FPI	Women reported higher depressive symptoms than men, with depression in women associated with ethnicity, clinic visit frequency, and social and sexual anxiety, whereas men’s depression was mainly linked to treatment cost pressure and social anxiety.
Benyamini et al. [39], 2009	Cross-sectional study	Israil	Sample 1 included 72 couples at their first visit to an infertility clinic and Sample 2 included 49 couples at various stages of treatment.	Infertility perception, distress and well-being	Illness Perception Questionnaire, Infertility-Specific Distress και Well-being Scales	Women had more negative views about infertility and perceived greater consequences than men, who generally maintained a more positive outlook.
Chiaffarino et al. [40], 2011	Longitudinal observational study	Italy	1000 consecutive couples	Depression and anxiety	ZDS and ZAS	Women showed greater anxiety and depression related to infertility than men, with specific psychosocial and clinical correlates for each gender.
Courbiere et al. [21], 2020	Prospective cross-sectional study	France	1045 French patients (355 men, 690 women) who were living or had lived the experience of infertility and medically ART	Emotional, psychosocial, relational, sexual, and work-related impacts of infertility and ART	Online survey with a 56-item author-developed questionnaire assessing psychosocial impact.	Women reported slightly greater psychological and physical burden than men, while both genders experienced comparable negative impacts on sexual life.
Donarelli et al. [41], 2012	Cross-sectional study	Italy	316 couples undergoing fertility treatments	Infertility-related stress	ECR Scale FPI STAI-S	Infertility-related stress was higher in women than men across most domains, except relationship concerns and acceptance of childlessness.
Gonen & Bokek-Cohen [24], 2020	Cross-sectional study	Israil	142 women and 62 men undergoing IVF	Emotional state	PANAS scale	Women reported significantly higher levels of negative emotions than men.
Ismail et al. [46], 2004	Comparative study	United Kingdom	30 couples for IVF	Depression and anxiety	MAACL questionnaire	Depressive symptoms peaked for both women and men before pregnancy testing, while anxiety levels remained relatively stable across IVF stages with no significant gender differences.
Lopes et al. [36], 2014	Cross-sectional study	Portugal	291 women and 92 men undergoing any stage of fertility treatment	QoL	-SCREENIVF -FertiQoL	Individuals at psychological risk showed significantly lower quality of life across all domains, with impairments more pronounced in women than in men.
McNaughton-Cassill et al. [56], 2002	Cross-sectional study	USA	Group 1: 26 couples that participated in the brief couples’ support groups Group 2: 19 other couples that did not attend the support group sessions and so comprised the control group	Depression, anxiety, optimism, irrational beliefs	BDI BAI LOT SPV SPS	Women showed reductions in anxiety and depression following participation, while men demonstrated increased optimism but also greater irrational beliefs; at baseline, women reported higher anxiety and depressive symptoms than men.
Onat & Aba [28], 2015	Cross-sectional study	Turkey	102 infertile women and 66 infertile men	Anxiety and health lifestyle	HPLP-II STAI	Women reported higher dispositional (trait) anxiety than men, while state anxiety levels were similar between genders; lifestyle behaviors and anxiety levels did not differ between pregnant and non-pregnant women.
Pottinger et al. [59], 2006	Cross-sectional study	Jamaica	51 couples prior to IVF beginning	Coping and psychological well-being	- Questionnaire for demographic characteristics and coping strategies. - GHQ-28	Women were more likely than men to report self-blame and emotional concealment related to infertility, while both genders commonly sought medical advice and expressed hope for spontaneous conception.
Rashidi et al. [31], 2008	Cross-sectional study	Iran	514 women and 514 men (n = 1028)	QoL	SF-36 short form	Men reported significantly higher health-related QoL than women across all physical, emotional, and mental health domains.
Schaller et al. [37], 2016	Prospective cohort study	Germany	119 women and 82 men undergo IVF treatment	Anxiety	STAI and 25 questions about stress factors	Women reported significantly higher state and trait anxiety levels than men.
Samorinha et al. [61], 2016	Cross-sectional study	Portugal	213 heterosexual couples undergoing fertility treatments	Anxiety and depression	STAI EPDS	Anxiety and depression levels were significantly higher in women compared with men.
Volgsten et al. [62], 2008	Cross-sectional study	Sweden	1090 consecutive women and men and 545 couples, attending a fertility clinic	Anxiety and depression	PRIME-MD	Psychiatric disorders were significantly more prevalent in women than men, driven mainly by higher rates of mood disorders and major depression, while anxiety disorder prevalence was similar across genders.
Volgsten et al. [50], 2010	Prospective study	Sweden	643 eligible women and men, 428 couples	Personality traits and psychiatric disorders	Swedish Universities Scales of Personality	Psychiatric disorders were more prevalent in women than men and were associated with higher neuroticism in both genders. In women, distress was linked to negative pregnancy outcomes, while in men it was associated with high neuroticism and male or unexplained infertility factors.
Winter et al. [34], 2016	Longitudinal prospective study	Belgium	185 women and 157 men (157 couples) with preimplantation genetic diagnosis or who conceive spontaneously or with ICSI.	Depression and anxiety	-EPDS -PRAQ -M/PAAS	Anxiety and depression did not differ significantly across conception groups.
Yassini et al. [52], 2005	Descriptive cross-sectional study	Iran	50 infertile couples who undergoing IVF (n = 25) or ICSI (n = 25) cycle	Depression and anxiety	Spielberger Anxiety Inventory BDI	Anxiety severity was higher among women receiving IVF or ICSI, whereas severe depression was more common among men undergoing ICSI.
Phromyothi et al. [53], 2003	Cross-sectional study	Thailand	60 infertile couples	Determinant factors of anxiety	Personal and Health Data Questionnaire, Cornell Medical Index, and the Determinant Factors of Anxiety	Most participants did not exhibit clinically significant emotional disturbance, with no significant gender differences in anxiety levels.
Boivin & Schmidt [54], 2005	Prospective, epidemiological cohort study	Denmark	818 couples	Infertility-related stress	Fertility Problem Stress Inventory, demographic and medical questionnaires, treatment evaluation.	Higher infertility-related stress predicted worse treatment outcomes for both genders, particularly among women, who exhibited greater anxiety.
Tome & Zwahlen [38], 2023	Cross-sectional study	International (including Australia)	1944 men and women with fertility problems	Mental health (including anxiety)	Mental health questionnaires, self-reports	A substantial proportion of participants reported mental health impact, with anxiety more frequently reported by primary patients than by partners.
Franco et al. [63], 2002	Cross-sectional study	Brazil	128 couples, that women have been at least 1 time an IVF/ICSI treatment	Psychological stress	PET-ART	High levels of anxiety were reported during IVF, particularly related to waiting for pregnancy test results, fear of negative outcomes, and concerns about repeated treatment, with women experiencing greater tension and anxiety than men.
Gonen & Bokek-Cohen [24], 2020	Cross-sectional study	Israil	142 women and 62 men undergoing IVF	Emotion state	PANAS scale	- Women reported significantly higher levels of negative emotions than men.
Holter et al. [45], 2006	Prospective, longitudinal study	Sweden	117 infertile couples in their first IVF treatment	Psychological well-being and marital relationship quality	PGWB index, a Questionnaire on the psychological impact of infertility (14-item scale) and Questions about the marital relationship.	Emotional responses during first IVF treatment were primarily determined by pregnancy outcome, with emotional well-being improving after successful treatment and worsening after treatment failure. Women reported stronger infertility-related emotional reactions than men, although men followed similar emotional trajectories when pregnancy was not achieved.
Madero et al. [27], 2017	Cross-sectional study	Spain	548 heterosexual individuals (347 women, 201 men) from Italy, Germany and France seeking IVF with donated oocytes in Barcelona, Spain	QoL, anxiety and depression	FertiQoL, HADS and Closed questions for psychological support	Men reported higher fertility-related QoL and lower anxiety than women, while significant cross-cultural differences were observed, with French individuals showing poorer QoL and greater need for mental health support compared with Italian and German participants.

Note: ART: Assisted Reproductive Technology; QoL: Quality of Life; SF-36: Short Form 36; RE: Role emotional; GH: general health; VT: Vitality; PHQ-9: Patient Health Questionnaire-9; FPI: Fertility Problem Inventory; SD: Standard deviation; IVF: In vitro fertilization; ZDS: Zung Depression Scale; ZAS: Zung Anxiety Scale; T1, T2 and T3: First, second and third assessment; CI: Confidence Intervals; ECR: Experiences in Close Relationships Scale; STAI-S: State scale of State-Trait Anxiety Inventory; PANAS: Positive Affect Negative Affect Schedule; MAACL: Mean Affect Adjective Check-List; PRIME-MD: Primary Care Evaluation of Mental Disorders; PRAQ: Pregnancy Related Anxiety Questionnaire; M/PAAS: Maternal/Paternal Antenatal Attachment Scale; ICSI: Intracytoplasmic Sperm Injection; VAS: Visual Analogue Scale; PET-ART: psychological evaluation test for assisted reproduction techniques; PGWB: Psychological General Well-Being; EPDS: Edinburgh Postnatal Depression Scale; HADS: Hospital Anxiety and Depression Scale; BDI: Beck Depression Inventory; BAI: Beck Anxiety Inventory; LOT: Life Orientation Test; SPV: Survey of Personal Views; SPS: Social Provisions Scale; HPLP-II: Health-Promoting Lifestyle Profile-II; GHQ-28: General Health Questionnaire.

**Table 4:** Studies with psychological reactions for those conceived with IVF.

Authors, Year	Design	Country	Sample Size	Psychological Outcomes Assessed	Materials and Methods	Main Results
Hjelmstedt et al. [44], 2003	Comparative study	Sweden	57 women pregnant after IVF and 55 male partners and 43 women who had conceived naturally (control group) and 39 male partners (control group)	Emotional state	IRS, Barnett scale, Karolinska Scales of Personality, Spielberger State and STAI	Compared with controls, women who conceived through IVF reported greater muscle tension and anxiety about pregnancy loss, while IVF men showed higher physical worry, indirect aggression, and guilt.
Repokari et al. [60], 2007	Prospective study	Finland	367 couples with singleton IVF/ICSI pregnancies	Psychological stress	DAS BDI short version socioeconomic status, stressful life events and infertility treatment characteristics questions	In women, depressive symptoms and stressful life events were associated with poorer couple functioning and marital satisfaction, particularly among ART women for stress-related effects, while men showed fewer relationship impacts, with stress affecting sexual satisfaction mainly in control men.
Stevenson et al. [32], 2019	Longitudinal, descriptive, pilot study	USA	22 IVF couples and 26 spontaneous conception couples	Stress and anxiety	PSS, STAI and PRAM	Anxiety levels, including pregnancy-related anxiety, were higher in women compared with men.
Hjelmstedt et al. [43], 2003	Longitudinal study	Sweden	57 women and 55 of their male partners who had conceived via IVF (experimental group) and 43 pregnant women and 39 of their male partners who had conceived naturally (control group)	Emotional responses and anxiety	IRS, ERPS and Wikman Attitude Scale	IVF couples reported higher anxiety about pregnancy loss throughout gestation; women experienced pregnancy more positively over time with decreasing loss-related concerns, while men showed increasing anxiety about the baby’s health and risk of injury during childbirth.
Gibson et al. [55], 2000	Prospective, controlled study.	Australia	IVF couples (65 women and partners) were compared with 61 age-matched controls without infertility.	Reports of general and parenthood-specific adjustment and attitudes to parenting.	-CES-D -EPDS -STAI -DAS -Intimate Bond Measure -PACR -Maternal Self-Efficacy Scale -Maternal Postnatal Attachment Questionnaire -Parenting Stress Index -Maternal Separation Anxiety Scale	Adjustment to parenthood was largely similar between IVF and control parents, although IVF mothers reported lower parenting confidence and IVF fathers lower marital satisfaction.

Note: BDI: Beck Depression Inventory; IRS: Infertility Reaction Scale; STAI: State scale of State-Trait Anxiety Inventory; DAS: Depression Anxiety Scale; PSS: Perceived Stress Scale; PRAM: Pregnancy-Related Anxiety Measure; CES-D: Center for Epidemiological Studies of Depression Scale; EPDS: Edinburgh Postnatal Depression Scale; PACR: Parenting Attitudes to Child Rearing Scale; ERPS: Emotional Responses to Pregnancy Scale.

**Table 5:** COVID-19-related and long-term IVF psychological reactions.

Authors, Year	Design	Country	Sample Size	Psychological Outcomes Assessed	Materials	Main Results
Barra et al. [22], 2022	Cross-sectional study	Italy	308 females and 216 males whose IVF treatments have been interrupted or postponed due to the COVID-19 pandemic	Depression and anxiety	GAD-7 PHQ-9	Women exhibited higher rates and severity of anxiety and depression than men.
Järvholm et al. [25], 2017	longitudinal study	Sweden	19 female and 17 males	Depression and anxiety	Semi-structured interviews and thematic analysis	Women experienced greater anxiety than men during IVF planning, with emotional distress normalizing after successful treatment.
Johansson et al. [9], 2010	Cross-sectional study	Sweden	36 men and 37 women in the unsuccessful IVF group (Group 1), 135 men and 154 women in the successful IVF group (Group 2) and 93 men and 118 women in the control group (Group 3).	Depression and anxiety	PGWB, SOC, experience of infertility questionnaire	Individuals in group 1 exhibited poorer psychological well-being than those in group 2, with men showing lower quality of life and sense of coherence, and women reporting higher levels of anxiety and depressive symptoms.
Johansson et al. [64], 2009	Cross-sectional study	Sweden	400 couples after unsuccessful IVF treatment	QoL	PGWB SOC VAS	Long-term psychological well-being was strongly associated with parenthood status, with individuals without children showing lower sense of coherence, higher depression, and reduced positive well-being. Women without children experienced greater psychological distress than men.
Filetto & Makuch [42], 2005	Longitudinal observational study	Brazil	92 couples, who had unsuccessfully undergone one or more IVF cycles were evaluated 3–8 years following their last failed attempt	Psychological patterns	Telephone interviews with semi-structured questionnaires	Women experienced greater emotional and identity-related burden than men, and treatment discontinuation was strongly associated with acceptance of infertility.

Note: IVF: In vitro fertilization; VAS: Visual Analogue Scale; PGWB: Psychological General Well-Being; SOC: sense of coherence; QoL: Quality of life.

## Data Availability

No new data were created or analyzed in this study. Data sharing is not applicable to this article.

## Supplementary Materials

The following supporting information can be downloaded at: https://www.mdpi.com/article/10.3390/healthcare14030375/s1, Table S1. Detailed EMBASE search strategy and results; Table S2. Detailed PubMed search strategy and results; Table S3. Detailed PsycINFO search strategy and results; Table S4. Detailed Scopus search strategy and results.
