# Supplementary material for: The Psychological Impact of In Vitro Fertilization (IVF): A Gender Systematic Review
Source: Healthcare (Basel). 2026 Feb 2;14(3):375. doi: 10.3390/healthcare14030375 (PMC12897396; doi:10.3390/healthcare14030375)
Supplement: Supplementary file 1 [file healthcare-14-00375-s001.zip › healthcare-4087686-supplementary.pdf]

Supplementary Tables

Table S1. Detailed EMBASE search strategy and results

Search	Query	Results
#1	'fertilization in vitro'/exp OR 'intracytoplasmic sperm injection'/exp	38798
#2	'fertilization in vitro':ab,ti OR 'in vitro fertilization':ab,ti OR 'test-tube fertilization':ab,ti OR (fertilization NEAR/3 'test tube'):ab,ti OR 'test-tube baby':ab,ti OR (bab* NEAR/3 'test tube'):ab,ti OR 'test tube babies':ab,ti OR (sperm:ab,ti AND (injection NEAR/3 intracytoplasmic):ab,ti) OR ivf:ab,ti OR 'intracytoplasmic sperm injection':ab,ti OR icsi:ab,ti	38754
#3	#1 OR #2	69789
#4	'psychology'/exp OR 'anxiety'/exp OR 'depression'/exp OR 'emotions'/exp OR 'psychological stress'/exp	564235
#5	('side effect' NEAR/3 psychological):ab,ti OR 'psychological side effect':ab,ti OR anxiet*:ab,ti OR depressi*:ab,ti OR emotion*:ab,ti OR distress*:ab,ti OR 'psychological stresses':ab,ti OR stress*:ab,ti OR (stress* NEAR/3 psychologic*):ab,ti OR 'mental suffering':ab,ti OR 'emotional stress':ab,ti OR psychosocial:ab,ti OR psycholog*:ab,ti	2459853
#6	#4 OR #5	3024088
#7	'infertility'/exp	943177
#8	'sterility reproductive':ab,ti OR sterility:ab,ti OR 'reproductive sterility':ab,ti OR 'sub-fertility':ab,ti OR subfertility:ab,ti OR infertil*:ab,ti	54084
#9	#7 OR #8	78117
#10	#3 AND #6 AND #9	814
#11	'randomized controlled trial'/exp OR 'controlled clinical trial'/exp OR random*:ab,ti OR placebo:ab,ti OR trial:ab,ti	917735
#12	#10 NOT #11	811
#13	'animal'/exp NOT 'human'/exp	4289942
#14	#12 NOT #13	713
#15	[2000–2023]/py	11586978
#16	#14 AND #15	525
#17	english:la	25484863
#18	#16 AND #17	479
#19	(man AND woman) OR (men AND women)	115
#20	#18 AND #19	79

Table S2. Detailed PubMed search strategy and results

Search	Query	Results
#1	"fertilization in vitro"[MeSH Terms] OR "sperm injections, intracytoplasmic"[MeSH Terms]	40801
#2	(((((((((((((Fertilization* in Vitro[Title/Abstract]) OR In Vitro Fertilization*[Title/Abstract]) OR Test-Tube Fertilization*[Title/Abstract]) OR (Fertilizations [Title/Abstract] AND Test-Tube[Title/Abstract])) OR (Fertilization* [Title/Abstract] AND Test-Tube[Title/Abstract])) OR Test Tube Fertilization*[Title/Abstract]) OR Test-Tube Bab*[Title/Abstract]) OR (Bab* [Title/Abstract] AND Test-Tube[Title/Abstract])) OR Test Tube Babies[Title/Abstract]) OR (Sperm Injection* [Title/Abstract] AND Intracytoplasmic [Title/Abstract])) OR IVF[Title/Abstract]) OR (Injection* [Title/Abstract] AND Intracytoplasmic Sperm [Title/Abstract])) OR Intracytoplasmic Sperm Injection*[Title/Abstract]) OR (Injections [Title/Abstract] AND Sperm Intracytoplasmic [Title/Abstract])) OR ICSI[Title/Abstract]	10537
#3	#1 OR #2	44040
#4	((((Psychology[MeSH Terms]) OR Anxiety[MeSH Terms]) OR Depression[MeSH Terms]) OR Emotions[MeSH Terms]) OR Stress, Psychological[MeSH Terms]	675549
#5	(((((((((((((((((Side Effect *[Title/Abstract] AND Psychological [Title/Abstract])) OR Psychological Side Effect*[Title/Abstract]) OR Anxiet*[Title/Abstract]) OR Depressi*[Title/Abstract]) OR Emotion*[Title/Abstract]) OR Distress*[Title/Abstract]) OR Psychological Stresses[Title/Abstract]) OR Stress*[Title/Abstract]) OR (Stress* [Title/Abstract] AND Psychologic*[Title/Abstract])) OR Psychologic* Stress*[Title/Abstract]) OR (Stress*[Title/Abstract] AND Life[Title/Abstract])) OR Mental Suffering[Title/Abstract]) OR (Suffering[Title/Abstract] AND Mental[Title/Abstract])) OR Suffering[Title/Abstract]) OR Emotional Stress[Title/Abstract]) OR (Stress [Title/Abstract] AND Emotional[Title/Abstract])) OR psychosocial[Title/Abstract]) OR psycholog*[Title/Abstract]	489164
#6	#4 OR #5	1015806
#7	Infertility[MeSH Terms]	73417
#8	((((((Sterility [Title/Abstract] AND Reproductive [Title/Abstract])) OR Sterility[Title/Abstract]) OR Reproductive Sterility[Title/Abstract]) OR Sub-Fertility[Title/Abstract]) OR Subfertility[Title/Abstract]) OR Infertil*[Title/Abstract]	96394
#9	#7 OR #8	123472
#10	#3 AND #6	1021
#11	((((((("randomized controlled trial"[Publication Type]) OR "controlled clinical trial"[Publication Type]) OR "ramdomized"[Title/Abstract]) OR "ramdomised"[Title/Abstract]) OR "placebo"[Title/Abstract]) OR "sham"[Title/Abstract]) OR "randomly"[Title/Abstract]) OR "trial"[Title/Abstract])	1540460
#12	#10 NOT #11	910
#13	(animals[MeSH Terms] NOT (humans[MeSH Terms] AND animals[MeSH Terms]))	5123697
#14	#12 NOT #13	901
#15	("2000"[Date - Publication] : "3000"[Date - Publication])	22188381
#16	#14 AND #15	640
#17	English[Language]	30928356
#18	#16 AND #17	591
#19	(((man) AND (woman)) OR (men)) AND (women)	361416
#20	#18 AND #19	66

Table S3. Detailed PsycINFO search strategy and results

Search	Query	Results
#1	MJSUB.EXACT.EXPLODE("Infertility")	1545
#2	ti(sterility NEAR/3 reproductive) OR ti(Sterility) OR ti("Reproductive Sterility") OR ti(Subfertility) OR ti(Sub-Fertility) OR ti(Infertil*)	1243
#3	ab(sterility NEAR/3 reproductive) OR ab(Sterility) OR ab("Reproductive Sterility") OR ab(Subfertility) OR ab(Sub-Fertility) OR ab(Infertil*)	2713
#4	#1 OR #2 OR #3	2969
#5	MJSUB.EXACT.EXPLODE("Reproductive Technology")	1225
#6	ti(Fertilization* in Vitro) OR ti((In Vitro Fertilization* OR IVF)) OR ti((Test-Tube Fertilization* OR Injection* NEAR/3 Intracytoplasmic Sperm)) OR ti((Intracytoplasmic Sperm Injection* OR Injections AND Sperm NEAR/3 Intracytoplasmic)) OR ti((Fertilization* NEAR/3 Test-Tube OR ICSI)) OR ti(Test Tube Fertilization*) OR ti(Test-Tube Bab*) OR ti(Bab* NEAR/3 Test-Tube) OR ti(Test Tube Babies) OR ti(Sperm Injection* NEAR/3 Intracytoplasmic)	241
#7	ab(Fertilization* in Vitro) OR ab((In Vitro Fertilization* OR IVF)) OR ab((Test-Tube Fertilization* OR Injection* NEAR/3 Intracytoplasmic Sperm)) OR ab((Intracytoplasmic Sperm Injection* OR Injections NEAR/3 Sperm NEAR/3 Intracytoplasmic)) OR ab((Fertilization* NEAR/3 Test-Tube OR ICSI)) OR ab(Test Tube Fertilization*) OR ab(Test-Tube Bab*) OR ab(Bab* NEAR/3 Test-Tube) OR ab(Test Tube Babies) OR ab(Sperm Injection* NEAR/3 Intracytoplasmic)	617
# 8	#5 OR #6 OR #7	1432
#9	MJSUB.EXACT.EXPLODE("Clinical Psychology")	6184
#10	MJSUB.EXACT.EXPLODE("Anxiety")	41101
#11	MJSUB.EXACT.EXPLODE("Depression (Emotion)")	18154
#12	MJSUB.EXACT.EXPLODE("Emotions")	194940
#13	MJSUB.EXACT.EXPLODE("Psychological Stress")	6223
#14	ti(Anxiet*) OR ti(Depressi*) OR ti(Emotion*) OR ti(Distress*) OR ti(Stress*) OR ti(Suffering) OR ti(psychosocial) OR ti(psycholog*)	388452
#15	ab(Anxiet*) OR ab(Depressi*) OR ab(Emotion*) OR ab(Distress*) OR ab(Stress*) OR ab(Suffering) OR ab(psychosocial) OR ab(psycholog*)	970789
#16	#9 OR #10 OR #11 OR #12 OR #13 OR #14 OR #15	1038956
#17	#4 AND #8 AND #16	298
#18	MJSUB.EXACT("Random Sampling")	183
#19	MJSUB.EXACT("Clinical Trials")	702
#20	ti(placebo) OR ti(trial) OR ti(random*)	35244
#21	ab(placebo) OR ab(trial) OR ab(random*)	242150
#22	#18 OR #19 OR #20 OR #21	247282
#23	#17 NOT #22	254
#24	PY=2000-2023	171

Table S4. Detailed Scopus search strategy and results

Search	Query	Results
#1	TITLE-ABS-KEY("in vitro fertilization" OR IVF OR "test tube fertilization" OR "test tube baby" OR "test tube babies" OR "intracytoplasmic sperm injection" OR ICSI OR (sperm W/3 injection))	83826
#2	TITLE-ABS-KEY(anxiety* OR depression* OR emotion* OR distress* OR stress* OR psychosocial OR psychology* OR "emotional stress" OR "mental suffering")	1596528
#3	TITLE-ABS-KEY(infertility* OR sterility OR "reproductive sterility" OR subfertility OR "sub-fertility")	47033
#4	#1 AND #2 AND #3	1254
#5	LIMIT-TO (LANGUAGE, "English")	1145
#6	LIMIT-TO (PUBYEAR > 1999 AND PUBYEAR < 2024)	862
#7	#4 AND #5 AND #6	797
#8	LIMIT-TO (DOCUMENT TYPE, "Atricle")	1033
#9	#4 AND #5 AND #6 AND #8	694
#10	(man AND woman) OR (men AND women)	115
#11	#4 AND #5 AND #6 AND #8 AND 10#	66
